# A Pilot Study of Salmon Protein Hydrolysate (ProGo^®^) Supplementation to Assess Its Impact on Hematologic, Integumentary and Metabolic Health

**DOI:** 10.3390/biomedicines14081663

**Published:** 2026-07-24

**Authors:** Crawford Currie, Tor Åge Myklebust, Christian Bjerknes, Bomi Framroze

**Affiliations:** 1 Hofseth BioCare, Keiser Wilhelms Gate 24, 6003 Ålesund, Norway; chbj@hofsethbiocare.no (C.B.); bf@hofsethbiocare.no (B.F.); 2 Department of Research and Innovation, More og Romsdal Hospital Trust, 6026 Ålesund, Norway; tor.age.myklebust@helse-mr.no

**Keywords:** salmon protein hydrolysate, bioactive peptides, iron metabolism, healthy ageing, inflammation, metabolic health

## Abstract

**Background:** Healthy ageing depends on the maintenance of several interconnected biological processes including adequate oxygen-carrying capacity, control of systemic inflammation, and preservation of lean body mass. ProGo^®^ is a salmon protein hydrolysate (SPH) with prior evidence of benefits on iron metabolism, inflammation, and metabolic health. **Methods**: This randomized, double-blind, active-controlled pilot study examined whether 56 days of daily ProGo^®^ supplementation could enhance key biological drivers of healthy ageing in overweight adults, compared with an iso-nitrogenous whey protein isolate (WPI). Primary outcomes were hematological parameters and a composite integumentary (hair, nail, and skin) score, assessed with Holm–Bonferroni multiplicity adjustment. Secondary outcomes included change in body composition, inflammatory biomarkers and glucose metabolism. **Results**: ProGo^®^ showed significant improvements in hemoglobin, RBC count, and ferritin, and in the integumentary composite score. Secondary analyses showed reductions in percent body fat and BMI, with preservation of lean body mass (LBM), alongside reductions in pro-inflammatory cytokines, HbA1c, and fasting blood glucose. WPI had minimal impact on any of these outcomes. **Conclusions:** ProGo^®^ supplementation improved multiple key biological drivers of healthy ageing: erythropoietic function, systemic inflammation, glycemic control, and body composition. These findings are consistent with a bioactive rather than a purely nutritional effect and are hypothesis-generating with respect to a multi-domain profile relevant to healthy ageing and the maintenance of intrinsic capacity. Confirmation in larger studies is warranted.

## 1. Introduction

The ageing of the global population has unsurprisingly been mirrored by a rising interest in longevity and wellbeing. Ageing is associated with a number of changes that include a gradual and progressive increase in inflammation within the body, termed inflammaging [1]. This appears to be driven by a number of factors and the net effect is inflammation-related damage to the body with an increased risk of cardiovascular disease, metabolic dysfunction and cancer along with other chronic diseases [2,3]. Ageing also sees a progressive decline in muscle mass, increased adiposity and reduced energy levels, the latter likely multifactorial including less efficient glucose metabolism, oxidative stress and a reduction in mitochondrial function and density [2,4].

Iron is an essential micronutrient required for oxygen transport, mitochondrial respiration, and multiple iron-dependent enzymatic processes central to cellular energy metabolism. Adequate iron availability is necessary for hemoglobin synthesis and erythropoiesis, thereby supporting efficient oxygen delivery and metabolic function [5]. Disturbances in iron homeostasis can impair red blood cell production and oxygen transport capacity with associated fatigue, reduced physical performance, and impaired concentration or cognitive function, including in some individuals without overt anemia [6]. Recent reviews indicate that iron deficiency remains the most prevalent micronutrient deficiency worldwide and continues to represent a major global health burden [7].

Although iron deficiency anemia represents the most clinically recognized manifestation of inadequate iron status, milder disturbances in iron metabolism may also have physiological consequences [8]. Suboptimal iron availability has been associated with reduced aerobic capacity, impaired immune function, and decreased metabolic efficiency, highlighting the importance of nutritional strategies that support iron metabolism and erythropoietic function. Traditional approaches to improving iron status have relied primarily on oral iron supplementation [9]; however, the clinical effectiveness can be limited by gastrointestinal intolerance [10], poor adherence, and the potential contribution of high luminal iron exposure to oxidative stress in the gastrointestinal tract [11]. Accordingly, there is interest in complementary nutritional strategies that may influence pathways relevant to iron handling or erythropoietic biology without acting primarily as a direct iron supplement [12].

The quality of the hair, nails and skin also declines noticeably with age, a result of both intrinsic and extrinsic factors. Increased levels of oxidative stress, such as from the regular consumption of processed foods and smoking, and inflammageing in general, will lead to an accelerated rate of damage to the dermal collagen and accelerated skin ageing reflecting underlying health challenges rather than merely an adverse aesthetic impact [1]. Dietary interventions, including supplementation, hold significant potential to improve health and support healthier ageing.

In this regard one emerging area of research focuses on bioactive peptides derived from food proteins which may exert physiological effects beyond their intrinsic nutritional value [12]. Protein hydrolysates produced through enzymatic digestion contain short peptide sequences that can interact with metabolic and cellular pathways [13]. Recent reviews highlight that food-derived peptides may exhibit diverse biological activities, including antioxidant and anti-inflammatory effects [14]. These peptides, typically composed of two to twenty amino acids, may exert biological effects through the modulation of cellular signaling pathways, redox balance, and nutrient metabolism.

Marine protein hydrolysates have received particular attention due to the unique amino acid composition and bioactive peptide profiles generated during enzymatic hydrolysis of fish proteins [15]. Systematic reviews of marine bioactive peptides report antioxidant, immunomodulatory, and metabolic effects in experimental models and emerging human studies [16]. The innate biological activity of these hydrolysates depends on both the protein source and the specific enzymatic hydrolysis process, which determines the resulting peptide profile and bioavailability.

ProGo^®^, a salmon protein hydrolysate (SPH), consists of a mix of peptides produced from Norwegian Atlantic salmon (*Salmo salar*) through enzymatic hydrolysis. This process yields a mixture of low-molecular weight peptides with high digestibility and bioavailability compared with intact dietary proteins.

ProGo^®^ has previously shown enhanced iron metabolism at a dose of 16g/day along with antioxidant and anti-inflammatory and apparent weight loss effects [17,18]. Experimental evidence points to ProGo^®^ containing DPP-IV, GLP-1, GIP and GLP-2 agonist activity [19,20]. Further work has isolated the peptides responsible for the iron modulatory effects via the upregulation of *FTH1* and ferritin, enhancing the iron storage capacity and correcting iron deficiency. The novel core amino acid sequence driving this action has been identified as EESGE [21].

In a 128-day open-label, single-arm proof-of-concept study, supplementation with a lower dose of ProGo^®^ (4 g/day) was associated with changes in perceived vitality, selected inflammatory markers, oxidative-stress-related readouts, and self-reported integumentary outcomes [22]. A supporting preclinical study of ProGo^®^ supplementation in aged rats demonstrated an improvement in cognitive function (learning and decision-making) accompanied by a reduction in gut dysbiosis and systemic and neuro-inflammation [23]. The 4 g/day clinical study lacked a control group, though the physiological effects of ProGo^®^ were consistent with those seen in previous trials, including those underlying the gene expression changes [24]. The consistent anti-inflammatory and antioxidant effects seen with ProGo^®^ across a range of doses could be expected to play a role in supporting overall health, including that of the skin, by protecting collagen from oxidative stress-related damage, such as from UV exposure.

Further preclinical assay-based research has shown potential for muscle health protection by inhibiting activin-A and myostatin, negative regulators of muscle mass [25,26], with a human equivalent dose of 11 g/d of ProGo^®^, similar to the 12 g/d used in this current study.

This randomized, double-blind, active-controlled pilot study evaluated whether supplementation with ProGo^®^ improved markers of iron metabolism, inflammation and metabolic health in overweight adults. The processes that erode healthy ageing, namely chronic low-grade inflammation, less efficient iron and glucose metabolism, muscle mass decline and the gradual accumulation of central adiposity, begin well before old age and are most amenable to modification when addressed during the adult decades in which they first emerge [2,4]. Overweight adults in early midlife therefore represent a physiologically relevant population in which to test whether a nutritional intervention can favorably influence these drivers before overt ill health develops.

Hematological and integumentary outcomes were selected as primary endpoints and markers of metabolic health, body composition and inflammation levels as secondary endpoints. We hypothesized, based on clinical and preclinical data, that daily ProGo^®^ supplementation would provide significantly greater improvements in outcomes than an iso-nitrogenous whey protein isolate comparator. The latter is assumed to provide only nutritional support and not additional bioactive health benefits.

## 2. Materials and Methods

### 2.1. Study Population

Healthy adult participants were recruited at a single clinical site run by Spectrum Clinical Research Ltd., Mumbai, India between July 2019 and January 2020 and the trial is registered on clinicaltrials.gov with the identifier NCT07616752. The protocol and all study-related documents were reviewed and approved by the ClinXXL Independent Ethics Committee, Mumbai, India, before the start of the study.

Eligible participants were aged 25 to 65 years with a body mass index (BMI) of 25.0–35.0 kg/m^2^. Participants were to be in good health based on self-declared medical history and a basic physical examination at screening. There was no requirement for participants to have established iron deficiency, anemia, or dermatological issues at baseline. Participants were asked to maintain their usual diet and level of physical activity throughout the intervention. However, dietary intake and physical activity were not formally recorded or quantified during the study.

All participants provided written informed consent and were required to comply with the study protocol, including supplement intake, scheduled visits, and follow-up. Key exclusion criteria included excessive physical activity, recent use of weight-loss medications, and clinically relevant medical conditions requiring treatment. Additional exclusions included active infection, pregnancy or lactation, known allergies to study ingredients, or any condition that could interfere with participation or data interpretation.

A total of 14 participants (10 females, 4 males) were randomized and completed the study with full compliance.

### 2.2. Study Design

This was an 8-week (56-day), single-center, sequentially randomized, double-blind, active-controlled clinical trial. The study was performed in accordance with the International Council for Harmonization Good Clinical Practice (ICH-GCP) guidelines.

Following screening, eligible participants attended a baseline visit (Day 0), at which they were randomized to ProGo^®^ or the whey protein isolate (WPI) comparator under double-blind conditions. Participants and study personnel responsible for conduct of the trial remained unaware of treatment assignment throughout the intervention period.

At baseline (Day 0) and at the end of the study (Day 56), participants underwent standardized assessments, including vital signs, anthropometric measurements (waist and hip circumference), and body composition analysis. Blood samples were collected for biochemical analyses, and participants completed a self-administered hair, nail, and skin health questionnaire. Participants were provided with identical, coded containers containing the study products, along with a standardized measuring scoop, to ensure maintenance of blinding throughout the study.

### 2.3. Interventional Product

The investigational product was ProGo^®^ (Hofseth BioCare ASA, Ålesund, Norway) a flavored salmon protein hydrolysate, produced via controlled enzymatic hydrolysis of filleted fresh Norwegian salmon to yield a mixture of low-molecular weight peptides.

ProGo^®^ is characterized by a high protein content (~99–100% by weight), low fat content (~0.3%), and low ash content (~2.3%), with >95% of the protein fraction present as water-soluble peptides. The degree of hydrolysis was approximately 10%, consistent with partial enzymatic hydrolysis.

Molecular weight distribution analysis shows that the peptide profile is predominantly composed of low-molecular weight fractions, with the majority of peptides <4000 Da, including substantial proportions within the 4000–2000 Da, 2000–1000 Da, and 1000–500 Da ranges.

The comparator consisted of a flavored whey protein isolate (WPI), matched for appearance, taste, and dose (12 g protein per serving), and administered using an identical preparation procedure to maintain blinding. Therefore, the trial was designed to compare ProGo^®^ with an iso-nitrogenous protein control (with equal calorific content) to assess the bioactive benefits of ProGo^®^ beyond purely nutritional effects. Both products were supplied in identical containers labeled with blinded codes.

Participants in the intervention group consumed 12 g of ProGo^®^ daily, administered as one scoop dissolved in approximately 150 mL of water and consumed within 15 min of preparation.

### 2.4. Biochemical Analyses

Fasting blood samples were collected at baseline (Day 0) and at the end of the intervention (Day 56) for assessment of hematological, metabolic, inflammatory, and safety biomarkers. Hematological parameters included hemoglobin and complete blood count (CBC) indices (hematocrit, red blood cell count, and mean corpuscular hemoglobin), along with serum ferritin, a marker of iron storage status. Metabolic markers included glycated hemoglobin (HbA1c) and fasting blood glucose (FBG). Serum biomarkers included interleukin (IL)-6, IL-8, IL-12 subunit β (IL-12B), tumor necrosis factor-α (TNF-α), zonulin, and ghrelin, which were quantified in serum using commercially available Multi-Analyte ELISArray kits (Qiagen GmbH, Hilden, Germany; distributed by Qiagen, Toronto, ON, Canada; catalog no. MEH-004A), which allowed simultaneous analysis of multiple protein targets. HbA1c was measured using the Tina-quant HbA1c Gen.3 immunoassay (Roche Diagnostics, Mannheim, Germany; catalog no. 05380026190). Fasting blood glucose was measured using GLUC3 Glucose HK Gen.3 assay (Roche Diagnostics, Mannheim, Germany; catalog no. 04404483190) and serum ferritin was measured using Abbott Laboratories’(Abbott Park, Chicago, IL, USA) Ferritin assay, catalog no. 05380026190. Serum samples were prepared by centrifuging whole blood at 1000 g for 15 min within 30 min of collection, followed by storage at −20 °C until analysis.

### 2.5. Instruments and Assessments

#### 2.5.1. Body Composition

Body composition was assessed using the InBody 570 multi-frequency bioelectrical impedance analysis (BIA) device firmware/software version 1.0 (InBody Co., Ltd., Cerritos, CA, USA), providing measurement output including body fat, fat-free mass, and related parameters. Anthropometric measurements included standardized waist and hip circumference. Multi-frequency BIA devices have been shown to provide reproducible estimates of body composition and demonstrate acceptable agreement with reference methods such as dual-energy X-ray absorptiometry (DXA) at the group-level, although individual-level precision may vary [27]. Vital signs assessed at each visit included blood pressure and heart rate, measured using standard clinical procedures.

#### 2.5.2. Self-Assessed Integumentary Health

The Hair, Nails, and Skin Self-Assessment Questionnaire consisted of seven items evaluating hair volume, hair softness, hair shine, hair strength, nail strength, skin hydration, and overall skin health. Each item was rated on a 6-point Likert scale ranging from 1 (“greatly satisfied”) to 6 (“greatly dissatisfied”), with lower scores indicating more favorable outcomes. The questionnaire was adapted from a previously developed instrument and administered at baseline and at Day 56.

### 2.6. Statistical Analyses and Sample Size Considerations

This study was designed as a pilot trial; therefore, no formal sample size or power calculation was performed. A total sample size of 14 participants was selected to assess feasibility, tolerability, and identify preliminary signals across relevant domains.

Continuous variables are presented as mean ± standard deviation (SD) at baseline and Day 56. Absolute changes from baseline are reported descriptively. Non-parametric Mann–Whitney and Fisher’s exact test were used to assess differences between groups at baseline.

Between-group differences at Day 56 were evaluated using linear regression models with the treatment group as a fixed effect and baseline values of the respective outcome included as a covariate. This approach was applied to both primary and secondary endpoints to account for baseline variability. Results are reported as estimated between-group differences with corresponding *p*-values. Complementary analyses using non-parametric Mann–Whitney test to test for differences in changes between groups were done to check if the results were dependent on model assumptions.

To address multiplicity among primary endpoints, the Holm–Bonferroni step-down procedure was applied across the six predefined primary outcomes: hemoglobin, RBC count, hematocrit, MCH, ferritin, and the composite hair, nail, and skin score. No correction for multiple testing was applied when analyzing secondary endpoints. All statistical analyses were performed using Stata MP version 18.0 (StataCorp LLC, College Station, TX, USA).

## 3. Results

### 3.1. Participant Flow and Baseline Characteristics

**Figure 1:**
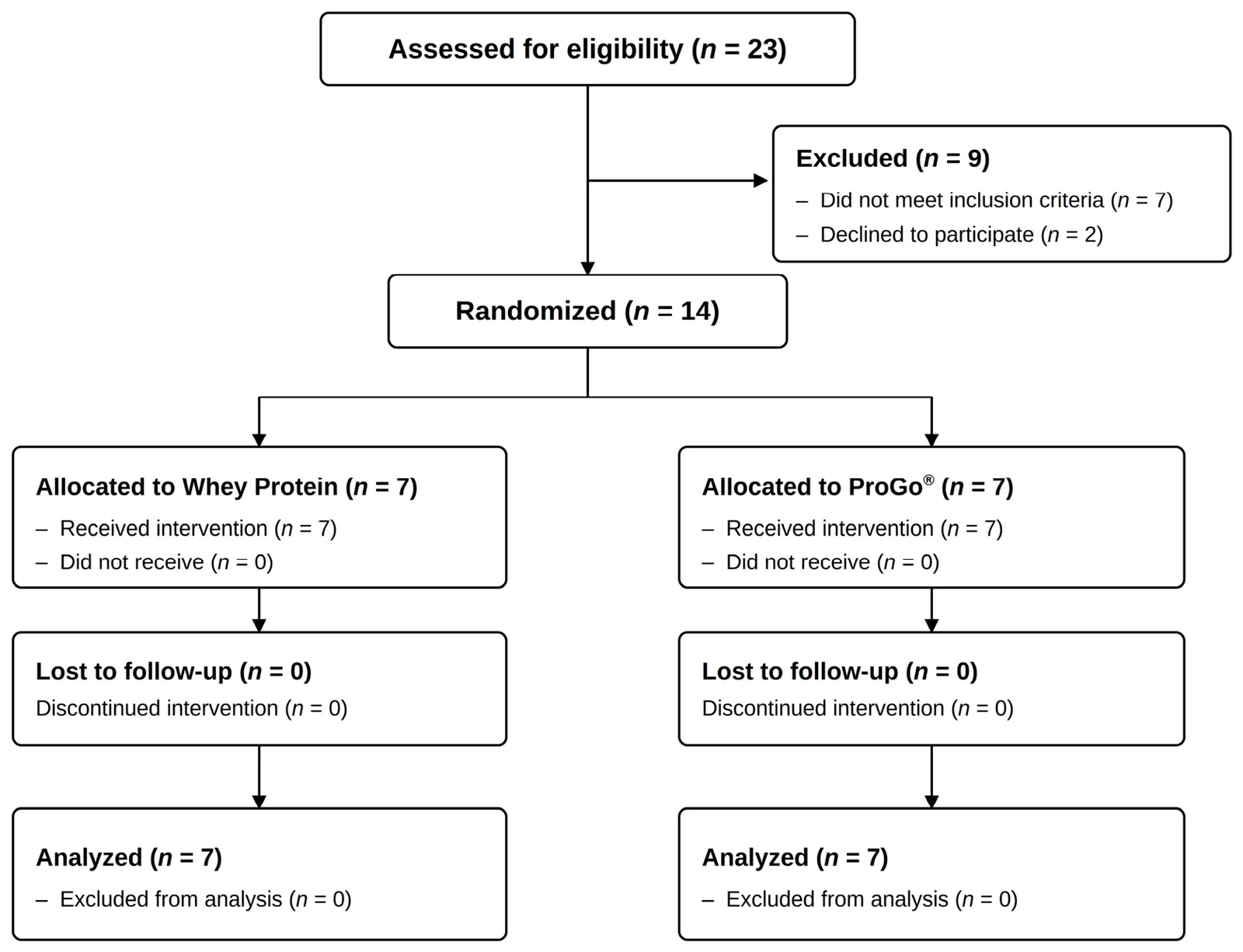
CONSORT flow chart showing enrollment, randomization, follow-up and analysis of participants.

A total of 14 participants were enrolled, randomized, and completed the study, with no dropouts (see Figure 1). All participants received their allocated intervention and were included in the analysis population. Compliance with study product consumption was 100%, as confirmed by returned container assessment. Participants were randomized 1:1, with seven assigned to the whey protein comparator group and seven to the ProGo^®^ group.

Baseline characteristics were broadly comparable between groups, including those for the primary endpoints, as presented in Table 1.

### 3.2. Primary Outcomes

#### 3.2.1. ProGo^®^ Significantly Increased Hemoglobin, RBC Count, and MCH Versus WPI

Primary outcome results are summarized in Figure 1. At Day 56, ProGo^®^ supplementation was associated with consistent differences across multiple hematological parameters relative to WPI. In baseline-adjusted analyses, between-group differences statistically favored ProGo^®^ for hemoglobin (+3.82 g/L; 95% CI: 0.70 to 6.95; *p* = 0.021), RBC count (+0.32 ×10^6^/µL; 95% CI: 0.19 to 0.46; *p* < 0.001), and MCH (+1.35 pg; 95% CI: 1.00 to 1.70; *p* < 0.001).

Within-group descriptive changes were directionally concordant, with mean increases in the ProGo^®^ group of 4.7 ± 3.0 g/L for hemoglobin (+3.8%), 0.33 ± 0.1 ×10^6^/µL for RBC count (+7.5%), and 1.5 ± 0.5 pg for MCH (+5.7%), with changes in the whey group being consistently smaller. Hematocrit increased in both groups (ProGo^®^ 1.9 ± 1.1%; whey: 0.6 ± 0.5%), but whilst the between-group difference favored ProGo^®^ (0.99%; 95% CI: −0.10 to 2.08) this did not reach statistical significance (*p* = 0.072).

Notably, the ProGo^®^ arm comprised a higher proportion of female participants (6 of 7; 85.7%) than the WPI arm (4 of 7; 57.1%) which might have impacted the primary hematologic endpoints. However, all hematological parameters were within the laboratory normal reference range at baseline with no significant differences between the groups.

#### 3.2.2. ProGo^®^ Significantly Increased Ferritin, Whereas WPI Produced No Change

The baseline-adjusted between-group difference in ferritin was 24.99 µg/L (95% CI: 15.18 to 34.81; *p* < 0.001), with a mean within-group increase of 24.6 ± 8.6 µg/L in the ProGo^®^ group and no meaningful change in the whey group (−0.4 ± 7.3 µg/L). In percentage terms, this equated to ferritin concentrations increasing by 13.4% in the ProGo^®^ group and remaining broadly unchanged (−0.2%) with the WPI comparator (see Figure 1).

#### 3.2.3. Primary Hematological and Integumentary Effects of ProGo^®^ Remained Significant After Holm–Bonferroni Adjustment

**Figure 2:**
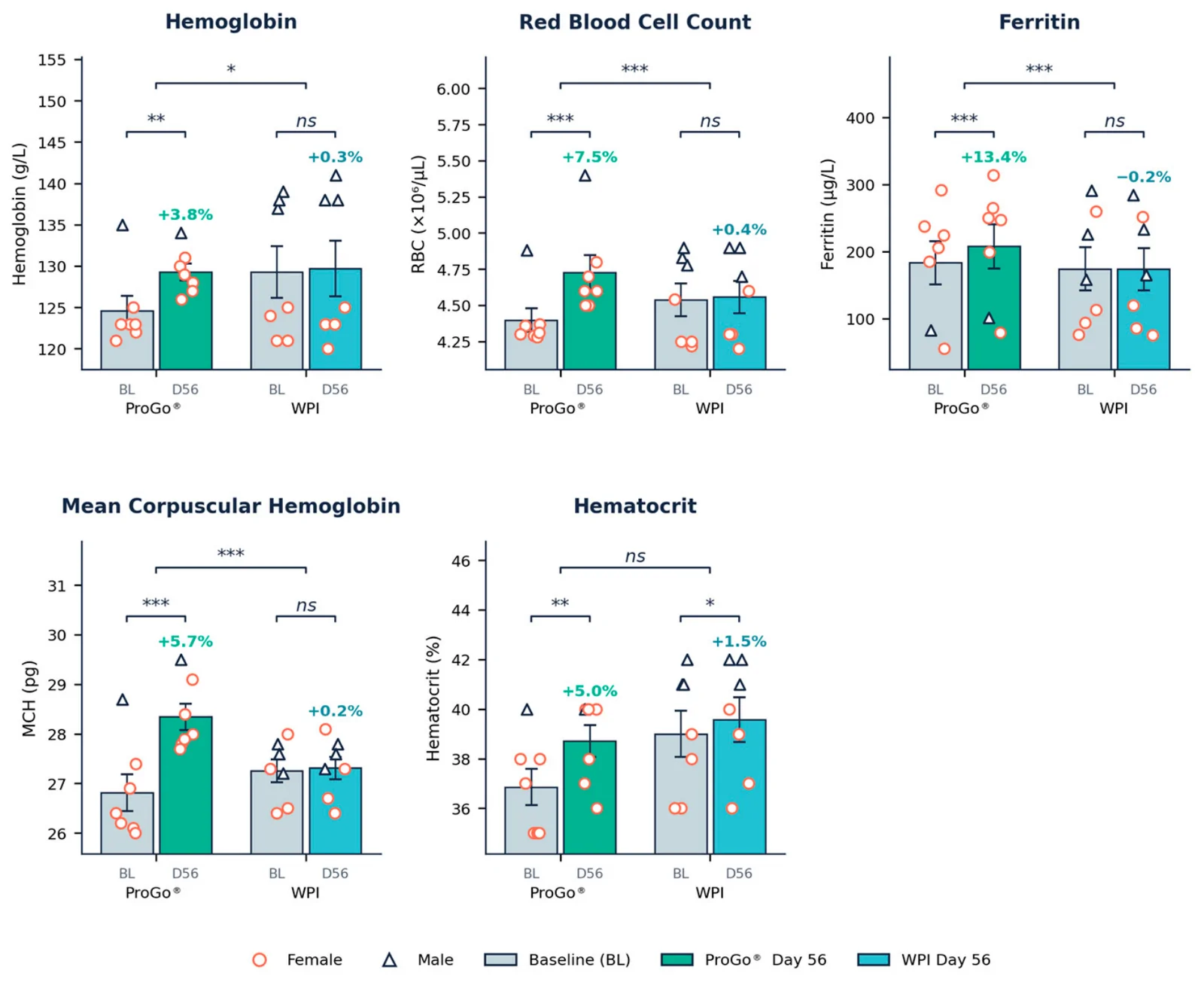
ProGo^®^ significantly improved primary hematological and iron-status outcomes versus WPI. Data are shown as dot–bar plots. Values are mean ± SD. Change represents percentage change from baseline (BL) to Day 56 (D56), with individual participant values overlaid. Female participants are shown as circles and male participants as triangles. Lower brackets indicate the statistical significance of the within-group change from baseline to Day 56 for each group and the upper bracket spanning the two groups indicates the between-group significance at Day 56. *p*-values are two-sided and Holm–Bonferroni multiplicity control was applied across the primary outcomes. Bold labels above each Day 56 bar give the mean percentage change from baseline. Each panel is headed with the analyzed parameter. The upper left panel displays the mean change in hemoglobin (g/L). The upper central panel displays the mean change in red blood count (RBC, ×10^6^/µL). The upper right panel displays the mean change in ferritin (µg/L). The left lower panel displays mean change in mean corpuscular hemoglobin (MCH, pg). The lower central panel displays the mean change in hematocrit (Hct, %). * Represents a significance level of *p* < 0.05; ** represents a significance level of *p* < 0.01; *** represents a significance level of *p* < 0.001; ns represents “not significant”. WPI represents whey protein isolate.

After Holm–Bonferroni adjustment across the six primary endpoints, the findings for hemoglobin, RBC count, MCH, ferritin, and the composite self-reported hair, nail, and skin score remained statistically significant (Figure 2 and Table 2). Hematocrit was not statistically significant before or after adjustment.

#### 3.2.4. Self-Reported Cosmetic Outcomes Favored ProGo^®^

The improvement in self-reported hair, nail, and skin quality, reflected by a decrease in the composite score, was noted in both groups. The ProGo^®^ group showed a larger reduction with a baseline-adjusted between-group difference of −1.62 (95% CI: −2.23 to −1.00; *p* < 0.001), indicating a greater subjective improvement. Mean within-group changes were −1.8 ± 0.7, equating to a relative 39.1% reduction in the ProGo^®^ group, and −0.3 ± 0.3, a relative 6.2% reduction, in the whey group.

Using non-parametric tests to compare change from baseline to Day 56 between groups on all primary outcomes gives very similar *p*-values. Notably differences in hematocrit values were significant using this test (*p* = 0.035), Supplementary Table S1, in contrast to the primary analysis.

### 3.3. Secondary Outcomes

Secondary outcomes are reported as nominal findings without multiplicity adjustment and therefore should be considered as hypothesis generating rather than confirmatory.

#### 3.3.1. ProGo^®^ Reduced Adiposity (Waist, Hip, BMI, Percent Body Fat) While Preserving Lean Body Mass

**Figure 3:**
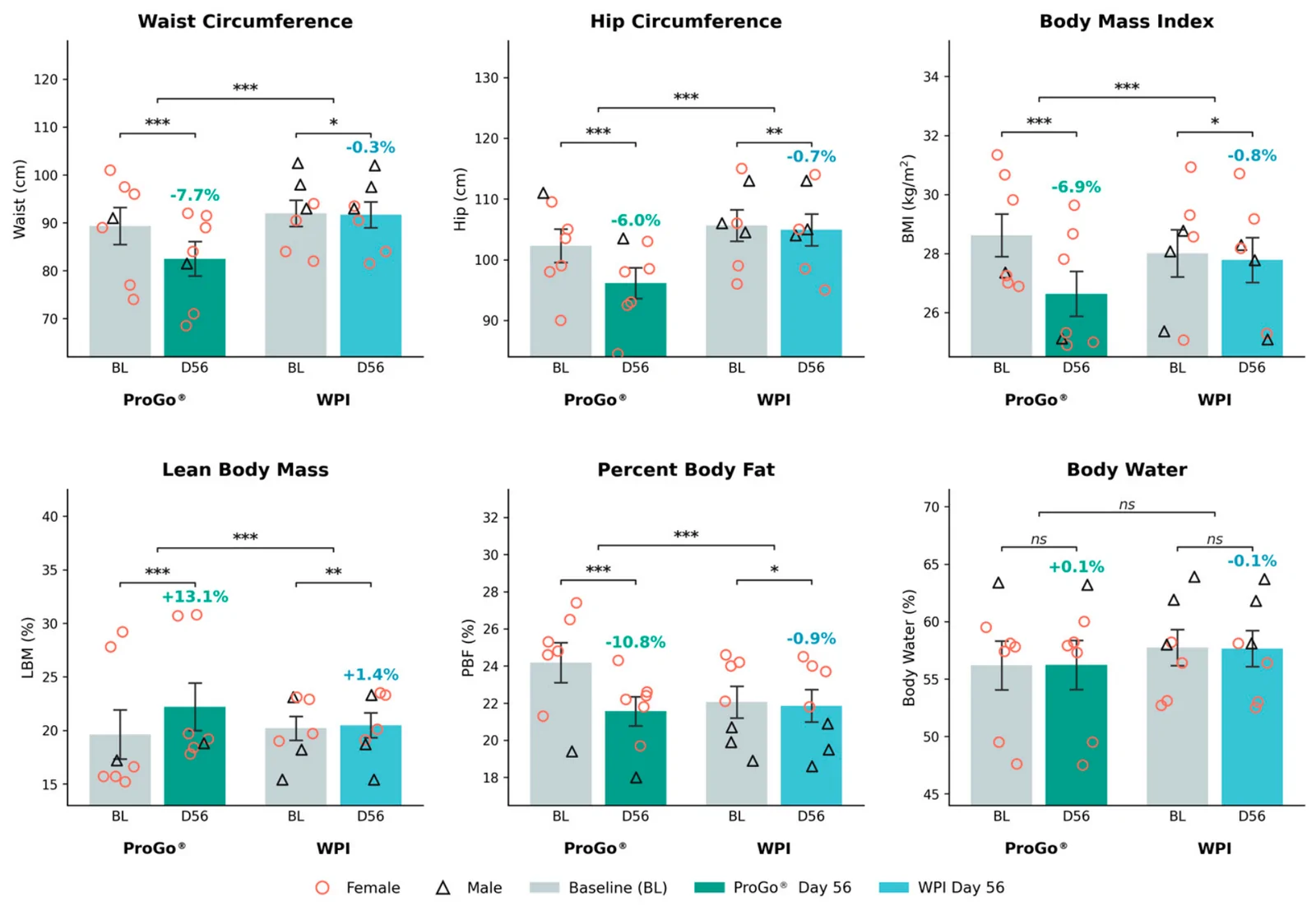
ProGo^®^ reduced adiposity and preserved lean body mass versus WPI. Data are shown as dot–bar plots: bars represent the group mean (±SD) at baseline (BL) and Day 56 (D56), with individual participant values overlaid. Female participants are shown as circles and male participants as triangles. Lower brackets indicate the statistical significance of the within-group change from baseline to Day 56 for each group and the upper bracket spanning the two groups indicates the between-group baseline-adjusted comparison at Day 56. Bold labels above each Day 56 bar give the mean percentage change from baseline. Change represents the relative percentage change from baseline. Between-group differences were estimated using baseline-adjusted linear regression. *p*-values are unadjusted for multiplicity. The top left panel shows the change in waist circumference; the top middle panel shows the change in hip circumference; the top right panel shows the change in body water; the bottom left panel shows the change in lean body mass; the bottom middle panel shows the change in percent body fat; and the bottom right panel shows the change in body mass index (BMI). Each panel is headed with the analyzed parameter. * Represents a significance level of *p* < 0.05; ** represents a significance level of *p* < 0.01; *** represents a significance level of *p* < 0.001 and ns represents “not significant”.

At Day 56, baseline-adjusted analyses showed between-group differences across several anthropometric and body composition variables, including waist circumference, hip circumference, percent body fat (PBF), BMI and lean body mass (LBM) (Figure 3). No between-group difference was observed for body water percentage, which remained unchanged in both groups (Figure 3). Absolute measurements and differences are provided in Table 3. Within-group changes from baseline to Day 56 were statistically significant in both groups for several body composition measures (Figure 3). The magnitude of change was substantially greater in the ProGo^®^ group as per the baseline-adjusted between-group comparison.

#### 3.3.2. ProGo^®^ Reduced Pro-Inflammatory Cytokines (IL-6, IL-8, IL-12B, TNF-α) Relative to WPI

At Day 56, baseline-adjusted analyses favored ProGo^®^ over the WPI comparator with lower mean concentrations observed for the inflammatory biomarkers IL-6, IL-8, TNF-α and IL-12B (see Table 4). Overall, the reductions in the ProGo^®^ group were around 2- to 10-fold greater than in the WPI group. There was minimal change in IL-17 in either group.

#### 3.3.3. ProGo^®^ Lowered Fasting Glucose and HbA1c Relative to WPI

**Figure 4:**
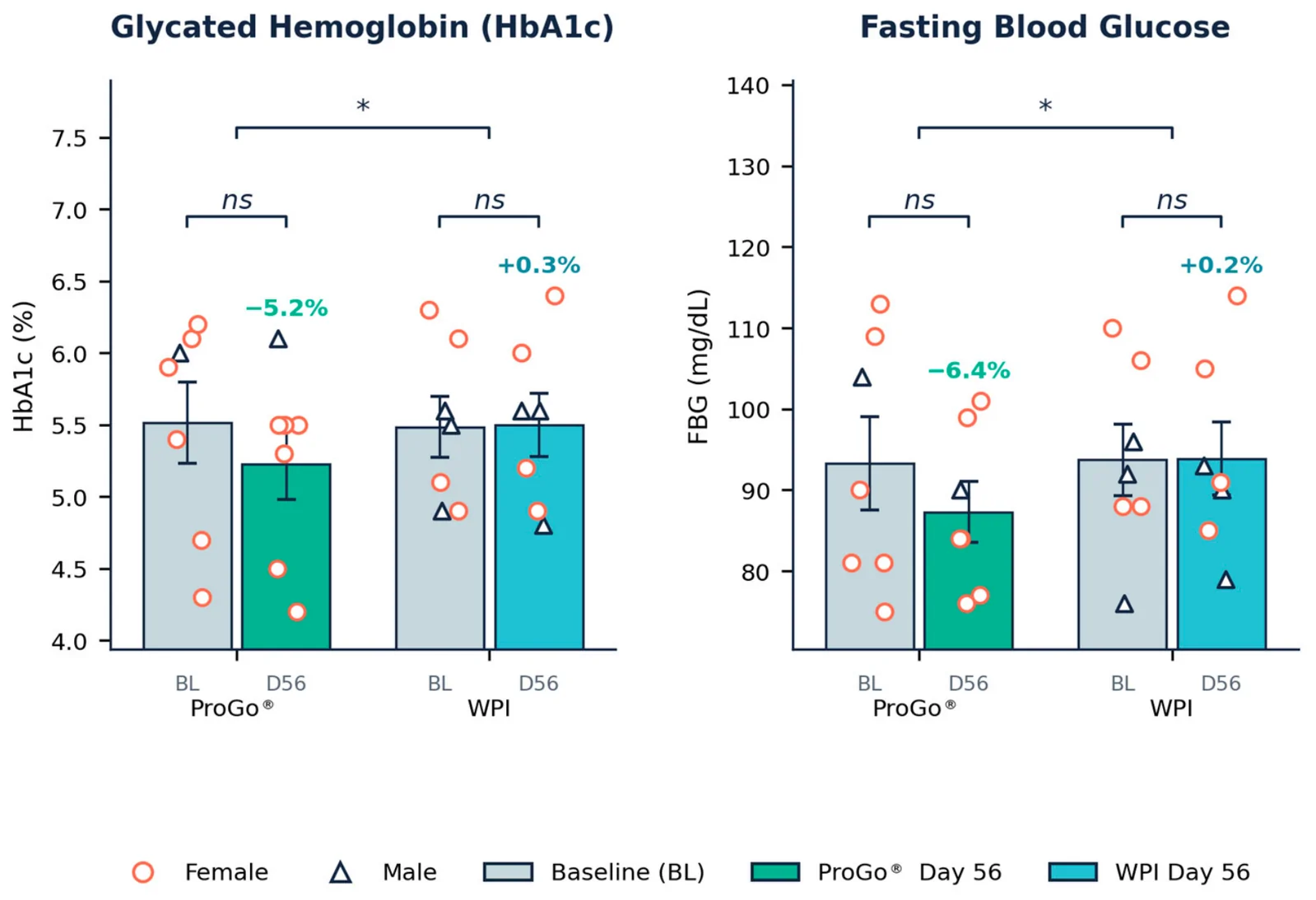
ProGo^®^ reduced fasting glucose and HbA1c versus WPI. Data are shown as dot–bar plots: bars represent the group mean (±SEM) at baseline (BL) and Day 56 (D56), with individual participant values overlaid; female participants are shown as circles and male participants as triangles. Lower brackets indicate the statistical significance of the within-group change from baseline to Day 56 (paired *t*-test) for each group; the upper bracket spanning the two groups indicates the between-group baseline-adjusted comparison at Day 56. Bold labels above each Day 56 bar give the mean percentage change from baseline. Each panel is headed with the analyzed parameter. Change represents absolute change from baseline with percentage change included in brackets for blood glucose changes. Between-group differences were estimated using baseline-adjusted linear regression. *p*-values are unadjusted for multiplicity. The left panel shows the changes in HbA1c and the right panel shows changes in fasting blood glucose. * Represents a significance level of *p* < 0.05; ns represents “not significant”.

At Day 56, baseline-adjusted analyses showed between-group favoring ProGo^®^ for fasting blood glucose (between-group difference −6.6 mg/dL (95% CI: −11.25 to −1.25); *p* = 0.019) and HbA1c (between-group difference −0.3%; (95% CI: −0.59 to −0.01); *p* = 0.045) when compared with WPI (which showed a change in HbA1c from baseline of 0% and −0.1 mg/dL for FBG) (Figure 4).

#### 3.3.4. Gut Barrier- and Hunger-Associated Biomarkers Zonulin and Ghrelin Showed No Significant Between-Group Differences

At Day 56, baseline-adjusted analyses showed no significant differences for zonulin or ghrelin. The mean decline in zonulin was 10.3% in the ProGo^®^ group and 2.7% in the WPI group (*p* = 0.120). Ghrelin showed a mean 5.9% decrease with WPI and a 1.8% decline in the ProGo^®^ group, which again was non-significant (*p* = 0.447).

Using non-parametric tests to compare change from baseline to Day 56 between groups on all secondary outcomes gives very similar *p*-values, as per Supplementary Table S1.

### 3.4. Safety Outcomes

#### 3.4.1. Clinical Safety and Adverse Events

Vital signs and clinical laboratory parameters remained within normal clinical ranges throughout the duration of the study and no clinically meaningful changes were observed between baseline and Day 56. Two adverse events consisting of mild gastrointestinal discomfort were reported by two participants during the study, with one event occurring in the WPI comparator group and the other in the ProGo^®^ group. Both events were considered unrelated to the study interventions and resolved within one week of onset. No serious adverse events were reported.

#### 3.4.2. Vital Signs and Clinical Chemistry

Consistent with previous clinical data, no meaningful changes were observed in systolic or diastolic blood pressure or serum creatinine in either group over the intervention period and all remained within their respective normal ranges. Heart rate was also essentially unchanged, with a small decline of 1.1 beats per minute (bpm) (±0.9) within the ProGo^®^ group, which was nominally significant when compared with baseline (*p* < 0.05) (Table 5).

## 4. Discussion

This randomized, double-blind, active-controlled study explored ProGo^®^’s health benefits in the context of healthy ageing, where the maintenance of iron homeostasis, the attenuation of low-grade inflammation, the preservation of metabolic efficiency, and the prevention of excess adiposity accumulation are increasingly recognized as determinants of intrinsic capacity, functional independence, and the avoidance of early frailty [28].

Over the 8-week supplementation period, ProGo^®^ was associated with an optimization of hematological parameters, including a significant increase in hemoglobin (+4%), red blood cell (RBC) count (+7%), and ferritin (+13%) compared with WPI. Notably, ProGo^®^ does not provide meaningful amounts of dietary iron, containing only 3.1 mg/kg and the putative mode of action is the 3-4-fold increase in *FTH1* noted in previous studies [22,24]. The comparator, WPI, had very limited effects on hematologic parameters and whilst it also contains very low levels of iron, the levels are higher (at 20 mg/kg) than in ProGo^®^. As a greater proportion of participants were female in the ProGo^®^ group, sensitivity analyses were performed on all primary outcomes when adjusting for sex in the regressions. The results remained largely unchanged with the exception of the differences in hematocrit values between ProGo^®^ and WPI, which became significant (*p* = 0.035).

Significant improvements were also seen in composite self-reported hair, nail, and skin scores, along with directionally consistent changes across the secondary analyses of inflammatory, metabolic, and anthropometric outcomes. WPI again had a very limited impact on these outcomes. In the context of the overall findings, it should also be noted that aesthetic outcomes not only indicate a potential to support perceived wellbeing but also provide a visible marker of underlying biological age and systemic health [1]. The self-reported integumentary findings also provide a complementary outcome signal consistent with iron’s role in collagen synthesis [29].

The maintenance of healthy hemoglobin and inflammation levels could help support an active lifestyle and potentially contribute to a reduction in the risk of frailty [30,31]. The significant reduction in inflammatory markers, IL-6, IL-8, IL-12 and TNF-α is consistent with previous research findings, including ProGo^®^’s upregulation of antioxidant protective pathways *FTH1*, *HMOX1* and *SOD1*, and the downregulation of oxidative stress-related genes including *ALOX12* [24]. *FTH1* encodes the heavy chain of ferritin, which has ferroxidase activity and can therefore store iron in the safer ferric form and protect against oxidative stress [32]. *HMOX1* and *SOD1* provide important antioxidant benefits for overall health as well as more specific actions, including supporting gut barrier function and metabolic health [33,34]. Elevations of *ALOX12* are seen in metabolic perturbations, including obesity, diabetes and metabolic syndrome [35]. These gene expression changes derive from earlier in vitro and preclinical work as well as a single-arm clinical study rather than from measurements made in the present trial and are noted as a plausible mechanism of action. Within the current study the combination of these effects could feasibly have contributed to a moderation of systemic inflammation, along with improved metabolic efficiency and reduced adiposity [35,36]. Indeed, given the role of IL-8 in the amplification of early inflammatory responses, the reduction seen with ProGo^®^ may be consistent with the attenuation of low-grade, chemokine-mediated inflammatory signaling in overweight individuals [36,37].

Improvements in glycemic markers were observed in parallel, including reductions in HbA1c (−0.30%, *p* = 0.045) and fasting blood glucose (−6.6 mg/dL, *p* = 0.019). While these changes could appear relatively modest in absolute terms, they emerged over a short intervention period (56 days) in healthy subjects. In the context of diabetes management, where a minimum decline in HbA1c of 0.5% is considered clinically meaningful, the reduction in this study for the maintenance of good health looks to be a potentially relevant finding [38]. Indeed, analysis of the landmark DCCT study indicated that a decrease in HbA1c of 0.3% would equate to only 13 diabetic patients needing to be treated to avoid the microvascular complication of retinopathy, demonstrating the likely relevance of this HbA1c change seen with ProGo^®^ in healthy adults [39]. An impaired glucose metabolism is a hallmark of advancing age and a key contributor to the elevated risk of type 2 diabetes, cardiovascular disease, and metabolic dysfunction. Therefore the maintenance of an efficient glucose metabolism is central to supporting energy availability, mitochondrial function, and the metabolic resilience that underpins healthy ageing [2].

Preclinical research suggests that the basis for ProGo^®^’s positive impact on glucose metabolism includes DPP-IV inhibition and an increase in direct glucose uptake into skeletal muscle cells [19]. However, cell line assays have also demonstrated that ProGo^®^ contains peptides that activate GLP-1 and GIP receptors as well as providing protective effects to the pancreatic islet cells [20]. These in vitro, cell line findings provide mechanistic plausibility for the glycemic effects observed in the present trial, although the relative importance of each has not been determined and other undiscovered actions could also interplay with the above mechanisms.

Consistent with previous clinical research, along with the mechanistic work described above relating to GLP-1 agonism, participants in the ProGo^®^ group showed significant improvements in body weight and body profile. Specifically, reductions relative to baseline in central and overall adiposity were observed alongside relative decreases in waist circumference (7.7%), hip circumference (6.1%), percent body fat (PBF) (−10.7%), and BMI (−7%). A relative reduction in BMI of at least 5% is considered clinically relevant in terms of positive underlying health benefits and this may have contributed to the anti-inflammatory effects noted in the ProGo^®^ group [36]. A reduction in central adiposity is considered most beneficial as it is more closely related to visceral fat reduction and a reduction of cardiometabolic risk [40]. Waist circumference is commonly used as a proxy of visceral adiposity and the reduction seen with ProGo^®^ appears clinically relevant. The body weight and body profile changes in the WPI group were limited, suggesting that ProGo^®^’s effect was not a nutritionally based effect. However, as diet and physical activity data were not collected, lifestyle change over the intervention period cannot be excluded as a contributing factor. Changes in the fasting levels of the orexigenic hormone ghrelin were also measured. The reductions in both arms were limited and there was no significant difference between the arms (*p* = 0.447). To put this into context, following gastric bypass surgery an over 70% reduction in ghrelin levels has been observed, whereas WPI and ProGo^®^ showed reductions of −5.9% and −1.8%, respectively [41]. Whether this numeric imbalance between the arms reflects WPI’s lower digestibility, as shown in a TIM-1 model, or merely natural variability in a small study is unclear [42].

Typically, weight loss is accompanied by a loss of skeletal muscle. Intriguingly, in this study the weight loss was the result of a reduction in PBF, with no reduction seen in the InBody-derived lean compartment. In terms of the lean compartment, there was no change in body water percentage, indicating that weight loss did not include a diuretic effect and that the weight loss was driven by fat loss. Maintaining the LBM has clear health implications including supporting physical function and reducing the risk of frailty, along with helping to maintain the basal metabolic rate [4].

The healthy weight loss profile of reduced adiposity and LBM preservation noted in this study is consistent with preclinical (in vitro and animal) research suggesting muscle protective activity by ProGo^®^ [22,23]. This includes the inhibition of the negative regulators of muscle mass, activin-A and myostatin, and a protection of human skeletal myoblasts in models of inflammation and cancer-driven muscle damage [22,23]. Whilst the interpretation of these findings requires caution with the small size of this study, the change in body profile and impact on wellbeing is of significant interest and is directionally consistent with related metabolic and inflammatory changes.

Ageing also sees a narrowing in the diversity and a change in the mix of the constituents of the GI microbiome with increased inflammation and a reduction in the GI barrier [2,43]. This results in the translocation of bacteria and lipopolysaccharides (LPS) contributing to systemic inflammation [44]. Preclinical work in aged rats and mice with induced bowel inflammation have shown that ProGo^®^ can restore the microbiome to a similar profile as a youthful one and reduce systemic inflammation [45]. In this study participants receiving ProGo^®^ showed a decline in the leaky gut biomarker zonulin of 10.3% compared with a 2.7% decline in the control group. Whilst this is an interesting signal, the difference was not significant, limiting the inferences that can be drawn from this result (*p* = 0.12).

When considered together, the findings in this study suggest a biological profile that could be relevant to maintaining intrinsic capacity. Intrinsic capacity is the composite of the physical and mental capacities of an individual, encompassing locomotion, vitality, sensory, cognitive, and psychological domains, and its maintenance across adulthood is a key aim for healthy ageing strategies [46]. The biological pathways engaged by ProGo^®^ in this study appear to potentially support the components of intrinsic capacity: erythropoiesis optimization, with likely enhanced oxygen delivery, and mitochondrial energy production; attenuation of inflammaging via multi-cytokine modulation; and metabolic resilience with improved glycemic and body profile composition with reduced adiposity and sustained LBM.

The trial participants were healthy although potentially at an early stage of metabolic risk from their weight profile. Indeed, this age profile reflects the study’s intent: the determinants of declining intrinsic capacity accrue across adulthood, and acting during the midlife window when inflammatory, metabolic, and body composition changes are emerging provides an opportunity to optimize the ageing trajectory in contrast to reversing established decline. Further, the cohort recruited to this study is highly prevalent in the global adult population and preventive nutritional strategies that have a lasting and meaningful impact are needed to improve longevity and wellbeing and reduce the risk of frailty. This study is a thought-provoking first step, but it is clear that larger studies of longer duration are needed.

This study has several limitations. It was a small (*n* = 14), exploratory trial and was not powered for confirmatory inference. The comparator was active rather than inert, which supports comparison against general nutritional-related effects but does not provide placebo-adjusted efficacy estimates. The integumentary outcome relied entirely on a subjective, self-administered satisfaction questionnaire, with no objective dermatological measurements. Self-reporting is susceptible to expectation and reporting bias, and this represents an important limitation of the integumentary findings. The InBody-derived lean compartment represents an indirect estimate of fat-free mass based on body water rather than a direct measure of skeletal muscle. Nevertheless, this is a validated method for assessing body composition changes, with good accuracy for estimating fat free mass in overweight subjects [47]. Secondary outcomes were numerous and analyzed without multiplicity correction, which introduces a risk of type I error. In addition, dietary intake, physical activity, and other lifestyle factors were not formally recorded during the intervention. These therefore represent potential unmeasured confounders, particularly for the body composition and glycemic outcomes. Care should therefore be taken when interpreting results. These results, along with the other endpoints, are nevertheless consistent with previous clinical trial and preclinical mechanistic research. The inclusion of an anti-inflammatory marker such as IL-10 could have provided insights into the change in the overall inflammatory balance and a marker such as adiponectin could have provided further insights into metabolic health. Further, the inclusion of measures, such as post-prandial glucose and insulin responses, would have helped to better characterize the glucose-regulatory profile of ProGo^®^ and garner further insights into the mechanisms of action driving this activity. This was, however, a pilot study and, despite these limitations, the trial also has strengths. These include randomized allocation, double-blinding, use of an iso-nitrogenous active comparator, complete retention, a high reported participant compliance, multiplicity control across the prespecified primary endpoints, and results consistent with previous randomized trials.

## 5. Conclusions

Healthy ageing is underpinned by a set of interconnected biological processes whose progressive decline drives metabolic vulnerability, frailty, and loss of functional independence. This randomized, double-blind pilot trial demonstrated that 56 days of daily supplementation with ProGo^®^, a salmon protein hydrolysate, at 12 g/day simultaneously improved several of these key biological drivers in overweight adults aged 25–65. Significant improvements in hemoglobin, RBC count, MCH, and ferritin, indicators of hemopoietic and erythropoietic function, were accompanied by a marked improvement in self-reported hair, nail, and skin quality. Concurrent secondary findings showed a favourable shift in body composition characterized by reduced adiposity with preservation of lean body mass, an attenuation of pro-inflammatory cytokines, and improved glycemic markers (HbA1c and fasting blood glucose).

The underlying mechanism of ProGo^®^ was not directly demonstrated in this trial and is inferred from prior clinical and preclinical evidence for ProGo^®^. Critically, however, none of these effects were observed in the iso-nitrogenous whey protein comparator group, suggesting a bioactive rather than a purely nutritional effect for ProGo^®^. Taken together, the breadth and internal consistency of these findings across multiple domains relevant to healthy ageing are noteworthy for a 56-day intervention in a small pilot cohort. Larger, adequately powered trials of longer duration are warranted to confirm these effects and to define the specific biological pathways through which ProGo^®^ exerts its multi-domain activity. Further, this trial work should include the analysis of serum metabolic profiles obtained via targeted metabolomics along with transcriptomic profiling (of white blood cells) to garner deeper insights into the beneficial effects of ProGo^®^.

## Figures and Tables

**Table 1:** Baseline participant characteristics (Day 0). Values are mean ± SD unless otherwise indicated. Values are mean ± SD unless otherwise indicated. Abbreviations: BMI, body mass index; RBC, red blood cell count.

Characteristic	ProGo^®^ (*n* = 7)	Whey (*n* = 7)	Total (*n* = 14)	*p*-Value
Female, *n* (%)	6 (85.7)	4 (57.1)	10 (71.4)	0.559
Male, *n* (%)	1 (14.3)	3 (42.9)	4 (28.6)	
Age (years)	46.0 ± 8.1	43.0 ± 8.0	44.5 ± 7.9	0.599
Weight (kg)	73.7 ± 12.5	71.2 ± 10.2	72.5 ± 11.0	0.805
BMI (kg/m^2^)	28.6 ± 1.9	28.0 ± 2.1	28.3 ± 2.0	0.0.805
Percent body fat (%)	24.2 ± 2.9	22.1 ± 2.3	23.1 ± 2.7	0.078
Hemoglobin (g/L)	124.6 ± 4.8	129.3 ± 8.3	126.9 ± 6.9	0.326
Ferritin (µg/L)	183.3 ± 85.2	174.0 ± 85.4	178.6 ± 82.1	0.949
Hematocrit (%)	36.9 ± 2.0	39.0 ± 2.4	37.9 ± 2.4	0.089
RBC (×10^6^/µL)	4.40 ± 0.2	4.54 ± 0.3	4.47 ± 0.3	0.971
Hair, Nail and Skin Composite Score	4.64 ± 0.7	4.8 ± 0.6	4.7 ± 0.6	0.691

**Table 2:** Primary hair, nail, skin (integumentary) outcomes (baseline, change, and between-group effects). Values are mean ± SD. Change represents absolute change from baseline to Day 56. Between-group differences are baseline-adjusted linear regression coefficients (β) with 95% CIs. *p*-values are two-sided. Holm–Bonferroni multiplicity control was applied across the six prespecified primary outcomes. WPI represents whey protein isolate.

Outcome	Group	Baseline	Day 56	Change (%)	Between-Group Difference (β [95% CI])	*p*-Value
Hair, nail, and skin composite score	WPI	4.8 ± 0.6	4.5 ± 0.7	−0.3 ± 0.3 (−6.2%)		
	ProGo^®^	4.6 ± 0.7	2.8 ± 0.6	−1.8 ± 0.7 (−39.1%)	−1.62 (−2.23 to −1.00)	<0.001

**Table 3:** Absolute changes in anthropometric and body composition outcomes. Values are mean ± SD. Change represents absolute change from baseline. Between-group differences were estimated using baseline-adjusted linear regression. *p*-values are nominal and unadjusted for multiplicity. WPI represents whey protein isolate.

Outcome	WPI Baseline	WPI Day 56	WPI Change	ProGo^®^ Baseline	ProGo^®^ Day 56	ProGo^®^ Change	Between-Group Difference (β, 95% CI)	*p*-Value
Waist circumference (cm)	92.0 ± 7.3	91.7 ± 7.2	−0.3 ± 0.3	89.4 ± 10.3	82.5 ± 9.5	−6.9 ± 1.7	−6.74 (−8.13 to −5.34)	<0.001
Hip circumference (cm)	105.6 ± 6.8	104.9 ± 6.9	−0.7 ± 0.4	102.3 ± 7.3	96.1 ± 6.7	−6.1 ± 0.7	−5.55 (−6.23 to −4.87)	<0.001
Body water (%)	57.7 ± 4.2	57.7 ± 4.2	−0.1 ± 0.1	56.2 ± 5.6	56.2 ± 5.7	+0.0 ± 0.2	0.13 (−0.09 to 0.36)	0.210
Lean body mass (%)	20.2 ± 3.0	20.5 ± 3.1	+0.3 ± 0.2	19.6 ± 6.1	22.2 ± 5.9	+2.6 ± 0.9	2.27 (1.51 to 3.02)	<0.001
Percent body fat (%)	22.1 ± 2.3	21.9 ± 2.3	−0.2 ± 0.2	24.2 ± 2.8	21.6 ± 2.1	−2.6 ± 0.9	−2.05 (−2.71 to −1.39)	<0.001
BMI (kg/m^2^)	28.0 ± 2.1	27.8 ± 2.0	−0.2 ± 0.2	28.6 ± 1.9	26.6 ± 2.0	−2.0 ± 0.2	−1.75 (−2.03 to −1.47)	<0.001

**Table 4:** Change in inflammatory biomarkers from baseline to Day 56. WPI represents whey protein isolate.

Outcome	WPI Baseline	WPI Day 56	WPI Change	ProGo^®^ Baseline	ProGo^®^ Day 56	ProGo^®^ Change	Between-Group Difference (β, 95% CI)	*p*-Value
IL-6 (pg/mL)	5.9 ± 1.6	6.2 ± 1.2	+0.3 ± 0.6	5.4 ± 1.9	4.7 ± 1.3	−0.8 ± 0.8	−1.26 (−1.86 to −0.67)	0.001
IL-8 (pg/mL)	22.0± 11.4	21.6 ± 9.8	−0.4 ± 2.1	25.6 ± 6.5	19.9 ± 4.2	−5.7 ± 3.3	−4.54 (−7.06 to −2.03)	0.002
IL-12B (pg/mL)	1.46 ± 0.4	1.44 ± 0.3	−0.02 ± 0.0	1.64 ± 0.3	1.47 ± 0.3	−0.17 ± 0.1	−0.14 (−0.21 to −0.06)	0.002
IL-17 (pg/mL)	13.5 ± 7.2	13.5 ± 6.9	−0.0 ± 0.8	14.2 ± 6.6	14.3 ± 7.1	+0.2 ± 0.6	0.16 (−0.66 to 0.98)	0.669
TNF-α (pg/mL)	20.3 ± 7.8	19.9 ± 8.4	−0.4 ± 1.1	19.0 ± 6.4	15.9 ± 4.3	−3.1 ± 2.5	−2.81 (−5.04 to −0.58)	0.018

**Table 5:** Safety outcome mean values ± SD at baseline and Day 56. Safety outcomes are presented descriptively; no formal between-group statistical testing was performed.

	ProGo^®^ Baseline	Day 56	WPI Baseline	Day 56
Creatinine (mg/dL)	0.79 ± 0.15	0.74 ± 0.10	0.70 ± 0.12	0.63 ± 0.11
Systolic blood pressure (mmHg)	123.7 ± 5.8	123.3 ± 6.3	129.2 ± 1.8	128.5 ± 2.2
Diastolic blood pressure (mmHg)	73.5 ± 4.3	72.3 ± 4.4	77.9 ± 3.0	77.7 ± 3.6
Heart rate (bpm)	74.3 ± 6.3	73.1 ± 6.7	77.0 ± 4.3	77.4 ± 4.3

## Data Availability

The original data presented in the study are openly available in FigShare at https://doi.org/10.6084/m9.figshare.32995358.

## Supplementary Materials

The following supporting information can be downloaded at: https://www.mdpi.com/article/10.3390/biomedicines14081663/s1, Table S1: Non-parametric sensitivity analysis of primary and secondary endpoints.

## Abbreviations

The following abbreviations are used in this manuscript:

ALOX12	Arachidonate 12-lipoxygenase
BIA	Bioelectrical impedance analysis
BL	Baseline
BMI	Body mass index
BPM	Beats per minute
CBC	Complete blood count
CI	Confidence interval
CONSORT	Consolidated Standards of Reporting Trials
D56	Day 56
DCCT	Diabetes Control and Complications Trial
DPP-IV	Dipeptidyl peptidase-IV
DXA	Dual-energy X-ray absorptiometry
FBG	Fasting blood glucose
FTH1	Ferritin heavy chain 1
GI	Gastrointestinal
GLP-1	Glucagon-like peptide-1
GLP-2	Glucagon-like peptide-2
GIP	Glucose-dependent insulinotropic polypeptide
HbA1c	Glycated hemoglobin
Hct	Hematocrit
HMOX1	Heme oxygenase 1
HSkMC	Human skeletal muscle cell
ICH-GCP	International Council for Harmonisation Good Clinical Practice
IL-	Interleukin-
LBM	Lean body mass
LPS	Lipopolysaccharide
MASCC	Multinational Association of Supportive Care in Cancer
MCH	Mean corpuscular hemoglobin
NF-κB	Nuclear factor-kappa B
PBF	Percent body fat
RBC	Red blood cell
SD	Standard deviation
SEM	Standard error of the mean
SOD1	Superoxide dismutase 1
SPH	Salmon protein hydrolysate
TNF-α	Tumor necrosis factor-alpha
TIM-1	TNO gastro-intestinal model-1
UV	Ultraviolet
WPI	Whey protein isolate
